# Patient and public involvement in the design and protocol development for a platform randomised trial to evaluate diagnostic tests to optimise antimicrobial therapy (PROTECT)

**DOI:** 10.3310/nihropenres.13591.1

**Published:** 2024-09-20

**Authors:** Martina Svobodova, Liza Keating, Melanie Gager, Cherry-Ann Waldron, Sammy Ainsworth, Julie Carman, Sarah Jones, Margaret Ogden, Graham Prestwich, PROTECT study team, Alasdair Corfield, Alasdair Gray, Andrew Tabner, Ashley David Price, Bethany Shinkins, Cat Atkin, Cherry-Ann Waldron, Clare Lendrem, Dan Lasserson, David Gillespie, Edd Carlton, Emma Thomas-Jones, Enitan Carrol, Graham Prestwich, Gurdeep Sagoo, James Wason, Jane O'Hara, Joanne Euden, Jonathan Sandoe, Jordan Evans, Julie Carman, Liza Keating, Luke Vale, Mandy Townsend, Margaret Ogden, Martina Svobodova, Nicola Howe, Paul Dark, Phil Pallman, Philip Howard, Raasti Naseem, Sarah Harle-Jones, Shalini Ahuja, Shrouk Messahel, Stacy Todd, Steve Bell, Tom Waterfield

**Affiliations:** 1 Centre for Trials Research, Cardiff University, Cardiff, Wales, CF14 4YS, UK; 2 Intensive Care Unit, Royal Berkshire NHS Foundation Trust, Reading, England, RG1 5AN, UK; 3 NIHR Clinical Research Facility Alder Hey, University of Liverpool, Liverpool, England, L12 2AP, UK

**Keywords:** patient and public involvement, platform trial design, inclusivity, clinical trial, inclusive patient and public involvement, underserved populations

## Abstract

**Background:**

Our patient and public involvement activities were part of a project aiming to develop a master protocol and National Institute for Health and Care research application for the PROTECT trial aiming to assess the effectiveness, implementation, and efficiency of antimicrobial stewardship interventions, to safely reduce unnecessary antibiotic usage by excluding severe bacterial infection in acutely unwell patients.

**Methods:**

Three public involvement sessions were held with representation from young people and parents, people from diverse backgrounds and people with experience of presenting to the emergency department with undifferentiated illness. The teleconference meetings lasted between 60-90 minutes, were recorded, notes were subsequently taken, and findings summarised. The data was collected on September 13, 2023, October 14, 2023 and February 28, 2024.

**Results:**

Working with public involvement contributors and public involvement groups at the protocol development stage provided an opportunity for the public to shape and influence the trial. We were able to establish the feasibility of the trial in the proposed setting and gain insights into how it would be perceived by potential trial participants. Antibiotic resistance was viewed as an urgent problem and research evaluating new technologies was deemed timely and important. The platform design was considered appropriate, time and cost-effective. Deferred and electronic methods of consent were viewed as acceptable if a clear and inclusive explanation is provided.

**Conclusions:**

Having access to public contributors with relevant lived experience was an important resource for the trial team. Identification and recruitment of public contributors via working with existing public involvement groups across the UK enabled the trial team to involve public members with varied life experiences and from diverse backgrounds. This project was a good practice example of how public involvement groups and practitioners across the UK can work together to deliver public involvement that is inclusive of relevant groups.

## Plain Language Summary

Our patient and public involvement activities were part of a project aiming to develop a master protocol for the PROTECT clinical trial aiming to evaluate several infection diagnostic tests to determine whether these improve care and patient safety, reduce use of unnecessary antibiotics, and provide value for money for the NHS.

Three public involvement sessions were held with representation from young people and parents, people from diverse backgrounds and people with experience of presenting to the emergency department with undifferentiated illness. The teleconference meetings lasted between 60-90 minutes, were recorded, notes were subsequently taken, and findings summarised. The data was collected on September 13, 2023, October 14, 2023 and February 28, 2024.

Working with public involvement contributors and public involvement groups at the protocol development stage provided an opportunity for the public to shape and influence the trial. We were able to establish the practicality of the trial in the proposed setting and gain insights into how it would be perceived by potential trial participants. Antibiotic resistance was viewed as an urgent problem and research evaluating new technologies was deemed timely and important. The platform design was considered appropriate, time and cost-effective. Deferred and electronic methods of participant consent were viewed as acceptable if a clear and inclusive explanation is provided.

Having access to public contributors with relevant lived experience was an important resource for the trial team. Identification and recruitment of public contributors via working with existing public involvement groups across the UK enabled the trial team to involve public members with varied life experiences and from diverse backgrounds. This project was a good practice example of how public involvement groups and practitioners across the UK can work together to deliver public involvement that is inclusive of relevant groups.

## Introduction

Clinical trials are the primary method for researchers to find out if a new drug, diagnostic test or vaccine is safe and effective. Meaningful and integrated patient and public involvement (PPI) is essential for researchers to gain insights from patients, their carers, families and the public, who have a moral right to be involved in research affecting them, and to ensure the quality and relevance of the research. PPI in research describes research which is “being carried out ‘with’ or ‘by’ members of the public” not just “‘to’, ‘about’ or ‘for’ them” and includes various activities and types of involvement across all stages of a study from design to dissemination ( https://www.invo.org.uk). A recent systematic review found that PPI is likely to improve participant recruitment levels for clinical trials ^1^.

The United Kingdom has become more ethnically diverse with 18.3% of England and Wales's population identifying as other than white in 2021, increasing by 8.7 percentage points since the 2001 Census ( Ethnicity Facts Figures). The percentage of people in Scotland with a minority ethnic background increased from 8.2% in 2011 to 12.9% in 2022 ( https://www.scotlandscensus.gov.uk). In Northern Ireland, the population identifying as ethnic minority - increased from 1.6% to 3.4% between 2011 and 2021 ( https://www.nisra.gov.uk).

The need for more inclusive practice and diversifying of PPI in trials has been increasingly recognised with respect to historically marginalised groups. The COVID-19 pandemic has amplified the conversation around the impact of health and social care inequalities on our society, where people from ethnic minority backgrounds were more likely to be infected and develop serious complications from the disease ^2^. The research system reflects and reinforces these inequalities through a lack of equitable access, diversity and inclusion across leadership, research teams, research participation, as well as public involvement.

In 2021 the National Institute of Health Research (NIHR) published surveyed patients and public involved with them as contributors to their work and research. The survey respondents were predominantly female (57%), 61 years of age and over, white and heterosexual ( NIHR Public Involvement Feedback). One of the practice recommendations coming out of the survey was to support and engage more diverse people with a range of knowledge, skills, and experiences to be involved in health research.

In this paper, we use the HRA Best Practice Principles for public involvement ( https://www.hra.nhs.uk/) in research to discuss inclusive public involvement when designing the PROTECT platform trial. With regards to our methodology, we focus on our decision-making around what involvement support was needed, who should be involved, what they should be asked to do, and what sort of lived experience or skills are relevant to this. The Guidance for Reporting Involvement of Patients and the Public (GRIPP2) short form checklist was used to guide the reporting of PPI in the paper ^3^.

### The PROTECT trial

Antibiotic resistance, where antibiotics no longer work against bacteria causing infections, could threaten the lives of millions of people around the world if urgent action is not taken. To prevent this problem, antibiotics should only be used for those patients who absolutely need them. When patients present to emergency care with a suspected infection, it is difficult for healthcare professionals to know if it is caused by bacteria (which needs treating with antibiotics) or a virus (which cannot be treated with antibiotics). There is no rapid test which can confirm bacterial infection, and current laboratory tests take too long to give results.

Clinicians are concerned about missing a diagnosis of sepsis, which is a potentially life-threatening complication of infection. The best treatment for sepsis includes early recognition, and prompt antibiotics and fluids delivered by a drip into a vein. The lack of a perfect test for bacterial infection, and the concern about delaying treatment for possible sepsis, leads to prescribing too many antibiotics.

There are new technologies which may help clinicians make decisions about whether to start antibiotics. They allow clinicians to identify which patients require admission to hospital and therefore avoid sending home patients who may get worse later if sent home. These technologies have never been evaluated in a large trial that allows multiple technologies to be tested, alone or in combination, wherever patients are seen with suspected infection. This form of study is called a platform trial, which allows faster decisions about which new tests should be used routinely, and improve patient safety. Platform trial designs have an established track record in infectious disease and have proved valuable in COVID-19 research ^4^.

The aim of the PROTECT (Platform Randomised evaluation of clinical Outcomes using novel TEChnologies to optimise antimicrobial Therapy) platform trial is to evaluate multiple technologies rapidly, and then adopt those that work quickly into care to benefit patients. We need to show that these technologies are safe for patients, improve care, and reduce the use of unnecessary antibiotics.

In the UK, a report commissioned by the UK Sepsis Trust estimated from Hospital Episode Statistics (HES) data an incidence of sepsis of 147,000 per year (inclusive of a further estimate of 10,000 children per year having sepsis). The majority of admissions and deaths with presumed bacterial infections occur in the elderly and those with comorbid disease ^5, 6^. Research shows a higher number of invasive infections in socially and economically deprived areas ^7, 8^. People with low socioeconomic status may be at a disadvantage compared to people with higher socioeconomic status: sepsis survival is greatly influenced by an individual’s underlying health status, their ability to recognise symptoms, and their access to optimal care at the right time.

### Aims

Funded by the Health Technology Assessment (HTA) Application Acceleration Award, this work was part of the overall project aiming to develop a master protocol and HTA application for PROTECT, a phase III, adaptive, multi-arm multi-stage (MAMS) platform trial to assess the effectiveness, implementation, and efficiency of biomarker-guided antimicrobial stewardship interventions, to safely reduce unnecessary antibiotic usage by excluding severe bacterial infection in acutely unwell patients.

PPI and stakeholder engagement was an important cross-cutting stream aiming to:

- Facilitate meaningful and inclusive public involvement in the PROTECT platform trial research development process by connecting the research team with existing public involvement groups
- Establish the feasibility and acceptability of the PROTECT study in the proposed setting
- Validate whether the research question was important to potential participants, whether the proposed methodology was acceptable and what outcomes were important to capture

## Methods

### Patient and Public Involvement

A PPI plan was designed in consultation with four lead PPI representatives who were involved in acquiring the funding for the PROTECT Acceleration Award, question development and research design. Three of these representatives were also included as public co-applicants in the HTA funding application, co-developing the lay summary and bringing different perspectives and experience to the development of the application and research.

The HRA four principles for meaningful involvement of patients and the public in health and social care research (Involve the right people, Involve enough people, Involve these people enough, Describe how it helps) are used below to describe our public involvement methodology:

**1. Involve the right people**

An NIHR equality impact assessment was conducted as part of the PPI plan development to ensure that the involvement process was inclusive and did not present barriers to participation or disadvantage to any groups affected by protected characteristics or other marginalising factors. Following the guidance from the NIHR INCLUDE project, we also considered the characteristics/demographics of the population which the PROTECT trial ultimately should serve, and established the need to focus on involvement of representatives from the following groups:

- People with lived experience of serious infection
- People presenting to the Emergency Department (ED) with undifferentiated illness
- Elderly people
- Children and young people
- Parents or carers of children under 16 years
- People from diverse ethnic minority communities
- People from disadvantaged socioeconomic backgrounds

**2. Involve enough people**

The trial team engaged with already existing public involvement groups to establish a trial-specific PROTECT PPI forum for ongoing consultation and involvement. Existing public involvement groups have established trusting relationships with members of the public, and people with a wealth of knowledge, diverse lived experience of health and social care services and have often received previous research training.

### Liverpool young people group

GenerationR Liverpool young people group is part of a National Network (GenerationR Alliance) of Young People’s Advisory Groups (YPAG) based across the UK. These groups are funded by the NIHR and/or other NHS organisations. The main remit of YPAGs is to support the design and delivery of paediatric research in the UK. The Liverpool YPAG group is made up of members who are aged between 8–19, parents and carers.

### Talking Trials group

The Talking Trials group is affiliated to the Centre for Trials Research, Cardiff University, as a diverse community-based advisory group providing input into the research development process within Centre for Trials Research. The group consists of 17 local community members of diverse ethnic minority backgrounds ^9^.

### Reading ED PPI group

The Reading ED PPI group is affiliated to the University Department of Emergency Medicine at the Royal Berkshire NHS Foundation Trust. It was established at the completion of the Quality Time Study. Quality Time used experience-based co-design as a quality improvement approach using participatory action research methodology ^10^. This enabled staff to work with patients and carers to work towards service improvement. The group initially focused on an ED volunteer programme but has developed to provide PPI input on both local research projects as well as the James Lind Alliance Emergency Medicine Priority Setting Partnership. It has a dynamic membership around a core group of twelve members.

**3. Involve those people enough**

During the development of the master trial protocol and the HTA funding application, three public involvement sessions were held with representation from young people and parents, people from diverse ethnic backgrounds and people with experience of presenting to the ED with undifferentiated illness. The meetings lasted between 60–90 minutes and were conducted via a teleconference. Sessions were recorded, notes were subsequently taken, and findings summarised. The YPAG consultation session took place on September 13, 2023. The PROTECT PPI forum comprising of both the Talking Trials and the Reading Quality Time public involvement groups took place on October 14, 2023, and February 28, 2024.

The YPAG session and the initial PROTECT PPI session used the same format and took place prior the HTA application stage 1 submission. Following the initial introduction of the PROTECT study proposal via a Power-point presentation, discussions focused on the proposed platform design; deferred and electronic consent; outcome measures and methods of testing; and translational sample collection. The second PROTECT PPI forum meeting took place as part of the HTA stage 2 application development process and focussed on providing feedback received from the funding committee from stage 1 and subsequently discussing this. Trial outcomes were presented and discussed by the group in terms of importance and relevance to patients.

**4. Describe how it helps**

Feedback from the successful stage 1 application together with an explanation how the group’s input contributed to this positive outcome, was conveyed to the YPAG group via the YPAG group facilitator at one of their regular meetings. The PROTECT PPI forum members discussed this stage 1 feedback during their second meeting in February 2024. The group was then invited to continue their involvement (pending the outcome of the stage 2 application) and be embedded in the trial research delivery, via regular meetings throughout the trial life cycle and governance, via being represented on the Trial Management Group (TMG).

## Results

The YPAG consultation session was attended by six young people aged 10–17, and three parents. The first PROTECT PPI forum session was attended by 22 people and the second by 35 people. The group demographics are shown in Table 1. The PPI forum attendees were provided with a £25 voucher per session (in line with the UK Standards for Public Involvement) to compensate for their time attending the meetings.

**Table 1.** PPI Forum attendees characteristics.

Demographics	PPI forum meeting 1 (n=22)	PPI forum meeting 2 (n=35)
Gender		
Female	12	24
Male	10	11
Ethnicity (self-defined)		
Asian	1	1
Bengali	n/a	1
Bissau-Guinean	1	1
Black British	n/a	1
Black Caribbean	n/a	1
British-Chinese	1	1
Chinese	n/a	1
Egyptian	1	1
English	2	2
Hindko	1	1
Indian	2	3
Nepali	n/a	2
Nigerian	1	1
Somali	n/a	2
Sudanese	2	4
Thai	1	1
Unknown	2	3
Welsh	1	1
Welsh Sikh	2	2
Welsh-Italian	1	1
White Asian	n/a	1
White British	3	3
Age		
<20	0	2
20-29	2	5
30-39	3	2
40-49	4	8
50-59	6	9
60-69	4	5
70-79	1	1
Unknown	2	3

### PROTECT trial aims and design

Antibiotic resistance was viewed as an urgent problem, and research evaluating new diagnostic technologies to support antibiotic prescribing decisions was deemed timely and important. The group felt more awareness raising was needed around antibiotic resistance and antibiotics overuse, so the general public understands the need for and the benefits of better diagnostic methods. The platform design was considered appropriate, cost-effective and time saving on the assumption that simple streamlined explanation is provided to potential trial participants including the potential risks and benefits of participation, whilst also making a clear distinction between diagnostic testing and treatments available when explaining the trial.

### Consent methods

Deferred method of consent was viewed as acceptable as long as the trial has a clear patient benefit and an explanation (i.e. justification for deferred consent) is provided. The group felt trial participants will understand the rationale behind deferred consent as long as there is reassurance that they will receive the best treatment option available. The use of electronic consent was also viewed as acceptable if offered via a study specific electronic device to avoid internet issues common in hospitals. The group also highlighted the need to consider the needs of diverse participant groups, and for example, offer paper-based information for elderly populations or information provided in minority languages for people not fluent in English.

### Outcome measures

The following outcomes were identified during the first PPI session as important to patients and were subsequently incorporated into the master protocol:

- Length of time to get the correct diagnosis
- Diagnostic test helping guide effective treatment
- Admission/re-admission to hospital or intensive care
- Use of antibiotics only for the period needed
- Reduction of side effects
- Length of stay in hospital
- Hospital re-admission with the same infection despite the test

The second PPI forum session re-considered both primary and secondary trial outcomes as listed in the draft master protocol. The group concluded that the trial would be successful if it helped establish the evidence for reliable diagnostic tests, and enabled clinicians to make timely decisions around treatment using appropriate antibiotics if clinically required (and when these can be stopped or changed). Other outcomes considered important were reduced mortality, reduced side effects, less time in both critical care and hospital, and improved health related quality of life.

## Discussion

Identification and recruitment of public contributors via working with existing public involvement groups across the UK enabled the trial team to involve public members with varied life experiences and from diverse backgrounds. Facilitating involvement requires careful consideration in order to be effective, responsible, and respectful to the PPI contributors. The trial team was able to utilise the PPI groups’ pre-established ways of working, and benefit from existing long-term trusting relationships amongst their group members.

The current UK research system, including the public involvement process, often lacks equitable access and under-served groups who are least included in health and social care research are not represented. This project was a good practice example of how the public involvement groups and practitioners across the UK can work together to promote diverse public involvement that is inclusive of relevant groups, ultimately making the process more transparent and shared.

## Conclusions

We were able to establish the feasibility of the trial in the proposed setting from a PPI perspective, and gain insights into how a platform trial design would be perceived by potential trial participants. Having access to public contributors with relevant lived experience was an important resource for the trial team. Involving a substantial number of PPI contributors at a very early stage provided the opportunity to shape and influence the trial design which will be instrumental when evidencing – to the funder as well as ultimately to the research ethics committee – that patient benefit, patient safety and wellbeing were a central and integral component of the PROTECT trial development and design.

The established PROTECT PPI forum will continue to influence the way the PROTECT trial is planned and carried out to improve the experience for people taking part in the trial. It will allow the trial team to respond quickly to changing research priorities and get the forum’s feedback on new trial arms as new experimental treatments are incorporated into the platform trial design. We will seek their insights on the diverse communication and information-giving practices. The group will co-design all our patient facing documentation including the video patient information and consent tool and will also pilot the translation tool in a variety of languages. They will also contribute to the development of a training video for recruiters that will also address any uncertainties and potential misperceptions when approaching potential trial participants not fluent in English.

## Data Availability

All underlying data are available as part of the article and no additional source data are required. Zenodo: GRIPP-2 checklist for ‘Patient and public involvement in the design and protocol development for a platform randomised trial to evaluate diagnostic tests to optimise antimicrobial therapy (PROTECT)’, https://doi.org/10.5281/zenodo.12162837 ^3^ Data are available under the terms of the Creative Commons Zero “No rights reserved” data waiver (CC0 1.0 Public domain dedication).
