# Supplementary material for: Patient and public involvement in the design and protocol development for a platform randomised trial to evaluate diagnostic tests to optimise antimicrobial therapy (PROTECT)
Source: NIHR Open Res. 2024 Sep 20;4:52. [Version 1] doi: 10.3310/nihropenres.13591.1 (PMC11736108; doi:10.3310/nihropenres.13591.1)
Supplement: Peer review history

# Reviewer report: Reviewer response for version 1

**DOI:** 10.3310/nihropenres.14753.r34316

**Published:** 2025-01-17

**Authors:** Emelda Eberechukwu

**Affiliations:** 1 Nigerian Institute of Medical Research, Yaba, Nigeria

The article described the use of Patient public involvement approach in the design and development of a protocol for a platform randomized trial to evaluate diagnostic tests to optimize antimicrobial therapy using existing public involvement groups across the UK. The project adhered to the HRA’s four principles for meaningful public involvement, ensuring robust participation and transparency in the research process and at the same time, demonstrated strong engagement with public contributors from diverse backgrounds. This inclusive approach enriched the trial’s design and relevance. The study design was purposeful with the trial focusing on reducing unnecessary antibiotic use which aligns with global priorities for antimicrobial stewardship, making its objectives impactful and timely. It would have been nice if further information was provided on the diagnostic tools that will be tested in the trial. For example, how many will be tested on this platform? What technologies are involved? The group unanimously agreed that diagnostic tools to support antibiotic prescribing decisions were timely and important.

 A few aspects of the study were a bit vague and would have benefitted from further clarification, for instance, although there was an apparent positive feasibility and acceptability of the platform, and the participants provided useful feedback in terms of overall platform design and desired outcome measure, It was however unclear if formal voting or structured deliberation processes were used to arrive at consensus on deferred consent, which might affect the robustness of the conclusions. The level of public involvement was a bit unclear, even though the authors mentioned that the PPI leads were involved in the initial proposal stage, it wasn’t clear if the public contributors were engaged at the initial stage. The exact stages of their involvement (e.g., proposal development, conceptualization) need clarification to assess the depth and impact of their participation.

 It is interesting to note that the PPI forum is amenable to differed method of consent and the use of electronic consent. However, the successful use of this method will largely depend on clearly communicating the rationale for deferred consent and ensuring consistent participant understanding of whichever consenting technique that is adopted. Overall, this is an interesting study with potential to improve antibiotic prescribing decisions and patient management and the adoption of a PPI approach will allow for continuous improvement over the lifespan of the project.

Is the work clearly and accurately presented and does it cite the current literature?

Yes

If applicable, is the statistical analysis and its interpretation appropriate?

Not applicable

Are all the source data underlying the results available to ensure full reproducibility?

Yes

Is the study design appropriate and is the work technically sound?

Yes

Are the conclusions drawn adequately supported by the results?

Yes

Are sufficient details of methods and analysis provided to allow replication by others?

Partly

Reviewer Expertise:

Infectious Diseases epidemiology and genetic basis of Antimicrobial resistance and Antimicrobial Stewardship programs using a One-Health approach

I confirm that I have read this submission and believe that I have an appropriate level of expertise to confirm that it is of an acceptable scientific standard, however I have significant reservations, as outlined above.

# Reviewer report: Reviewer response for version 1

**DOI:** 10.3310/nihropenres.14753.r34321

**Published:** 2025-01-15

**Authors:** Bashar Haruna Gulumbe

**Affiliations:** 1 Federal University Birnin-Kebbi,, Birnin Kebbi, Nigeria

Overall, this manuscript provides a coherent and logical exploration of how patient and public involvement (PPI) was integrated into the PROTECT platform trial design. It effectively highlights the benefits of engaging a diverse set of contributors early in the research process.

 I have the following observations:

1. While the manuscript describes the engagement of multiple existing PPI groups, it does not detail how their feedback was synthesized into actionable trial protocol changes. Offering a succinct summary of “feedback received” versus “adjustments made” (for instance, in a table or bullet points) would highlight the tangible impact of public input and strengthen the manuscript’s credibility.
2. The discussion looks shallow. The authors could expand the discussion by highlighting comparable examples from other platform trials that employed robust patient and public involvement practices, emphasizing what worked well and why. They might also address how future efforts could engage underrepresented groups not already affiliated with formal PPI networks. Finally, a more direct comparison with similar studies would underline the PROTECT trial’s unique contributions and reveal potential areas for refinement.

Is the work clearly and accurately presented and does it cite the current literature?

Yes

If applicable, is the statistical analysis and its interpretation appropriate?

Not applicable

Are all the source data underlying the results available to ensure full reproducibility?

Yes

Is the study design appropriate and is the work technically sound?

Yes

Are the conclusions drawn adequately supported by the results?

Yes

Are sufficient details of methods and analysis provided to allow replication by others?

Yes

Reviewer Expertise:

Microbiology, with a focus on antimicrobial resistance (AMR), antibiotic stewardship and genomics

I confirm that I have read this submission and believe that I have an appropriate level of expertise to confirm that it is of an acceptable scientific standard.

# Reviewer report: Reviewer response for version 1

**DOI:** 10.3310/nihropenres.14753.r33572

**Published:** 2024-12-14

**Authors:** Rasha Abdelsalam Elshenawy

**Affiliations:** 1 University of Hertfordshire, Hatfield, England, UK; 2 Consultant of Antimicrobial Resistance,, South Centre, Geneva, Geneva, Switzerland; 3 Pharmacy Development, FADIC, Harrow, Hertfordshire, UK

The manuscript "Patient and public involvement in the design and protocol development for a platform randomised trial to evaluate diagnostic tests to optimise antimicrobial therapy (PROTECT)" presents a thorough and well-documented account of the PPI process in the development of the PROTECT trial. The authors have successfully engaged a diverse range of public contributors, ensuring that the trial design is inclusive and reflective of the needs and perspectives of potential participants.

 The study design is robust, with clear objectives and a well-structured methodology. The detailed descriptions of the PPI sessions, including the demographics of participants and the feedback process, provide a comprehensive understanding of how public input was integrated into the trial design. The manuscript also highlights the importance of involving public contributors with relevant lived experiences, which is a significant strength of the study.

 The conclusions drawn are well-supported by the results, demonstrating the feasibility and acceptability of the trial design from a PPI perspective. The manuscript also discusses the broader implications of the findings, emphasizing the value of inclusive public involvement in clinical research.

 Overall, the manuscript is of high scientific standard and provides valuable insights into the role of PPI in clinical trial design. The authors have adhered to relevant reporting guidelines and have made all underlying data available, ensuring transparency and reproducibility.

Is the work clearly and accurately presented and does it cite the current literature?

Yes

If applicable, is the statistical analysis and its interpretation appropriate?

Not applicable

Are all the source data underlying the results available to ensure full reproducibility?

Yes

Is the study design appropriate and is the work technically sound?

Yes

Are the conclusions drawn adequately supported by the results?

Yes

Are sufficient details of methods and analysis provided to allow replication by others?

Yes

Reviewer Expertise:

NA

I confirm that I have read this submission and believe that I have an appropriate level of expertise to confirm that it is of an acceptable scientific standard.

# Response

**Published:** 2025-01-13

**Authors:** Martina Svobodova

**Affiliations:** Centre for Trials Research, Cardiff University, Cardiff, Wales, UK

Many thanks for providing your review for the article, this is very much appreciated.
